# Modelling and Differential Quantification of Electric Cell-Substrate Impedance Sensing Growth Curves

**DOI:** 10.3390/s21165286

**Published:** 2021-08-05

**Authors:** Anna Ronja Dorothea Binder, Andrej-Nikolai Spiess, Michael W. Pfaffl

**Affiliations:** 1 Animal Physiology & Immunology, School of Life Sciences Weihenstephan, Technical University of Munich, Weihenstephaner Berg 3, D-85354 Freising, Germany; Ronja.Binder@wzw.tum.de (A.R.D.B.); michael.pfaffl@wzw.tum.de (M.W.P.); 2 Center for Cardiology, Genomics and System Biology, UKE, D-20246 Hamburg, Germany

**Keywords:** ECIS (impedance vs. time), IPEC-J2 (adherent cells), segmented regression, four-parameter logistic, smoothing spline, area under the curve (AUC)

## Abstract

Measurement of cell surface coverage has become a common technique for the assessment of growth behavior of cells. As an indirect measurement method, this can be accomplished by monitoring changes in electrode impedance, which constitutes the basis of electric cell-substrate impedance sensing (ECIS). ECIS typically yields growth curves where impedance is plotted against time, and changes in single cell growth behavior or cell proliferation can be displayed without significantly impacting cell physiology. To provide better comparability of ECIS curves in different experimental settings, we developed a large toolset of R scripts for their transformation and quantification. They allow importing growth curves generated by ECIS systems, edit, transform, graph and analyze them while delivering quantitative data extracted from reference points on the curve. Quantification is implemented through three different curve fit algorithms (smoothing spline, logistic model, segmented regression). From the obtained models, curve reference points such as the first derivative maximum, segmentation knots and area under the curve are then extracted. The scripts were tested for general applicability in real-life cell culture experiments on partly anonymized cell lines, a calibration setup with a cell dilution series of impedance versus seeded cell number and finally IPEC-J2 cells treated with 1% and 5% ethanol.

## 1. Introduction

A large number of external and environmental influences, including drug treatments under laboratory conditions, exert a promoting or inhibiting effect on the overall growth of cell populations [1,2,3,4], which is typically represented and visualized as biological growth curves [5,6,7,8,9,10]. For the assessment of positive or negative influences on these populations, a comparison of growth curves derived from treated and non-treated control groups can be performed in these cases [11,12], and—if significant changes between groups are detected—quantitative estimation of this effect can be performed.

A common feature of growth curves is the partition into five essential and chronological subregions: (1) a flat baseline, either before any growth has started or on which growth occurs but is not detectable by the instrumental system due to the lack of sufficient sensitivity, (2) an exponential growth phase, (3) a subsequent linear growth phase, (4) a negative exponential transition phase (5) and finally the saturation or plateau phase in which growth is stalled [13]. Using these curves, it is possible to assess growth behavior using certain parameters as endpoints, such as the comparison of populations at one selected timepoint [14,15]. However, the use of single curve features for quantitation is prone to information loss as other characteristics of the curve are ignored; therefore, using the entire curve trajectory is more informative [8,16,17,18]. Along these lines, various methods have been established for the statistical evaluation of growth curves derived from the population observation of plants [19], animal species [17], microbial growth [18] and cell cultures [16].

For measuring the growth and proliferation of cell populations under laboratory conditions, different kinds of direct and indirect cell counting methods are available [20]. For this purpose, methods that can assess the confluence of cells without changing their environment (i.e., temperature and gas composition) and without moving cells are most desirable in order to enable observation of growth behavior that is more standardized and repeatable. In addition to life-imaging methods [21], indirect live monitoring via impedance measurement is important in this context [22,23]. If adherent cells are of interest, the electric cell-substrate impedance sensing system (ECIS) poses a viable option [22] as the growth of cell populations can be monitored in a simple and noninvasive manner [22], where the output is given as time versus impedance curves that resemble the classic growth curve partitions described above. While most evaluation methods for ECIS-generated growth curves use complex mathematical models to fit frequency [24] versus impedance [25,26], models that incorporate time usually do not directly employ impedance [25,27,28,29].

The aim of our project was to create R software scripts that enable a complete analysis pipeline for ECIS curves by analyzing impedance *versus* time growth curves consisting of the five subregions already mentioned. R scripts provide previously unavailable modules in which growth curves produced with the ECIS software can be imported as Excel .xlsx files (Microsoft, Redmond, WA, USA), combined, deleted, modified (e.g., normalized) and compared on the basis of specific curve locations. In the ECIS analysis, a large amount of data is created due to many timepoints being sampled. Therefore, and in order to minimize information loss, in our approach, the complete growth trajectory is exploited by fitting three different models to the impedance curve: a four-parameter logistic model (log) [30,31,32], a segmented regression model (seg) [33] and a smoothing spline model (spl), and the optimal fitting model for the respective cell line has to be chosen individually. Although these fitting routines are available to some extent in base R, they are not usable per se, as starting and smoothness parameters have to be automatically calculated and transferred to the fitting functions.

For subsequent statistical analysis, a plethora of fit parameters and curve reference points are extracted that allow conclusions from changes in growth behavior, e.g., of a treatment group in comparison to a control group. Particularly noteworthy are the first and second derivative maxima (*y* (impedance) and the corresponding *x* (time) values), the *x* value at 10%, 20% or 50% of the maximum increase in impedance and the nodal points of the segmented regression model. These selected parameters are largely found in the existing growth model implementations such as qPCR quantification [34,35] and a range of bioassay applications [36], but needed to be implemented anew as R does not provide automatic reference points extraction from fitted curves.

The R scripts were developed on the basis of the ECIS growth curves generated with porcine jejunal epithelial cells (IPEC-J2, RRID:CVCL_2246) and ECIS model 1600 (Applied Biophysics, US, Troy). The scripts were subsequently tested on the IPEC-J2 cells treated with ethanol in the (sub)lethal range (1% and 5% EtOH). In addition, a dilution series was analyzed for the same cell line to correlate the seeded cell number with the results of the script and impedance values. In order to test a greater variety of ECIS curve types and trajectories (flat, steep, variable plateaus), four known and two unknown (anonymized) cell lines were also interrogated.

## 2. Materials and Methods

### 2.1. Cell Culture, Information and Growing Conditions of the Used Cell Lines

IPEC-J2 cells were cultured under 5% CO_2_, 37 °C and 95% air humidity, and all sterile work was performed using a lamiar flow bench (ENVAIR eco, Emmendingen). The cells were cultured with DMEM/F12 (Gibco, Thermo Fisher Scientific, without HEPES, with glutamine, phenol red, Schwerte), 5% FCS (Sigma, Hamburg), 100 U/mL penicillin/streptomycin (Sigma, Hamburg, Germany), tested negative in PCR and DAPI staining for *Mycoplasma* spp. (PCR: AppliChem, PanReac, PCR Mycoplasma Test Kit, Darmstadt; DAPI: Pierce DAPI, Thermo Fisher Scientific, Karlsruhe, Germany) and only used in one of the passages 1–4 after thawing.

**Figure 1:**
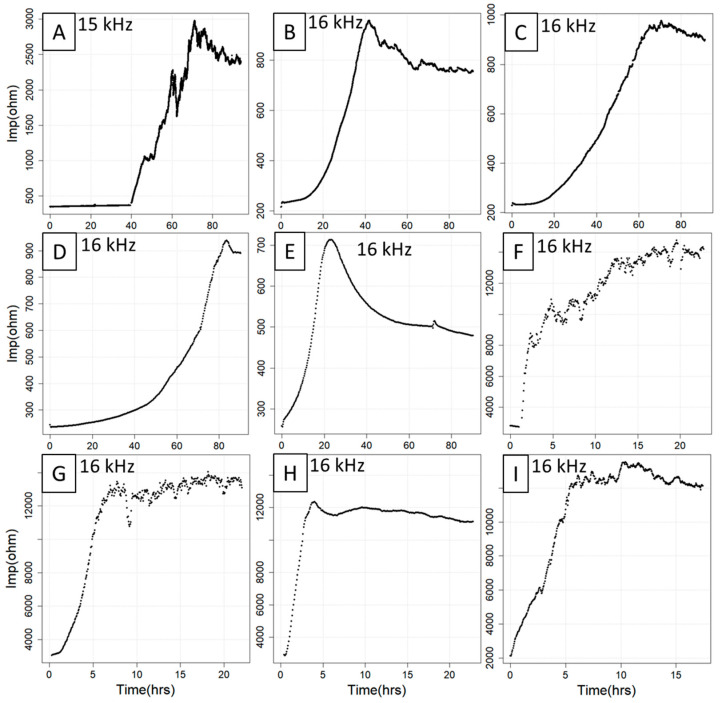
Compilation of nine ECIS curves with different features and trajectories. The curves were obtained from seven experiments involving IPEC-J2 (**A**), CACO2 (**B**,**C**), C2BBe1 (**D**,**E**), HUVEC (**F**,**G**), MDCK cell lines (**H**) and an unknown cell line (**I**) under different treatment regimes. Note that the curves vary substantially with respect to baseline length (**A**,**B**,**E**), curve smoothness (**A**,**E**,**F**), slope (**C**,**E**,**F**) and plateau length and linearity (**D**,**E**,**G**–**I**). hrs = hours, Imp = impedance.

All the experiments with other cell lines that were used for the verification of the script and for Figure 1 were partially anonymized so that no details about the cultivation and treatment of the cells can be given. All the experiments involving non-IPEC-J2 cells were carried out at a different laboratory with different equipment, and the data were handed over partially anonymized. These data were kindly provided by Ibidi (Germany, Gräfelfing) and Applied Biophysics (US, Troy).

### 2.2. Impedance Measurement and General Experimental Settings

The electric cell-substrate impedance sensing system (ECIS) consists of an 8- or 96-well cell culture dish with 1–40 gold electrodes embedded at the bottom of the well [22]. If gold electrodes become covered with cell bodies, the impedance increases in a way that results in a growth curve divided into five subregions described in the “Introduction” [13]. A change in the impedance level can be generated in various ways: enlargement, change in shape, migration, micromotion and division of individual cell bodies [27,37,38] and, finally, cell–cell connections [38]. Thus, an increase in impedance and the corresponding growth curve are always multifactorial so that a statement about the overall growth behavior without further investigations into the individual cell line can only be made for an entire population. At the plateau phase, it can be assumed that ~100% confluency is reached and a homogeneous epithelial layer is formed; however, it cannot be excluded that further changes such as cell duplications affect overall impedance.

For all experiments with IPEC-J2 cells, a single testing frequency of 15,000 Hz was employed, while for the anonymized cells, multiple frequency testing was chosen. The experiments were performed (ECIS model 1600, Applied Biophysics, US, Troy) on 8-well plates with 10 gold electrodes each (8W10E PET, Ibidi, Gräfelfing, Germany). The ECIS 8-well plates were incubated with the cell culture medium for around three days to generate a stable individual baseline that was further measured for at least 12–24 h. For all the IPEC-J2 experiments (except for the cell dilution experiments), the cells were seeded with the density of 10,000 cells per 400 µL medium per well with the growth area of ~0.8 cm² (25,000 cells/mL and 12,500 cells/cm², respectively). For the seeding procedure, the cells were added to the end volume of the preheated (37 °C) cell culture medium. Inversion of the cell–medium mixture before seeding was performed to ensure homogenous dispersion of the cells in the wells. The 8-well plates with seeded cells were incubated for around 20 min at room temperature (RT) to enable the cells to attach to the electrode surface. Subsequently, the plates were fastened in the ECIS plateholder in an incubator (HERACELL VIOS 250i, Fisher Scientific, Schwerte) at 37 °C, 5% CO_2_ and 95% humidity and the ECIS system was started. In all the experiments except for the cell dilution experiments, on each 8-well plate, two wells were used purely as the medium control group (mCG) without cells, and two wells were used as the cell control group (control group, CG), where only cells with the medium but without treatment were added. For the dilution experiments, no mCG were performed.

### 2.3. IPEC-J2 Dilution Experiments and Cell Counting on ECIS Dishes

To draw better conclusions about the impedance changes of the ECIS growth curve in relation to the type of growth behavior of the specific IPEC-J2 cell population, a cell dilution series was performed on ECIS 8-well plates (8W10E PET, Ibidi, Gräfelfing). In three independent experiments, 100,000, 75,000, 56,250, 42,188, 31,641, 23,731, 17,798 and 13,348 cells were seeded in duplicates (Supplemental Data 2, “Cell Counts”; *n* = 6). The impedance was measured individually in every well with the ECIS system until an endpoint was reached (Supplemental Data 2, “Curve Data”). The endpoints were set as follows: the first one was chosen in the plateau phase so that the epithelium was formed after ~22 h (*n* = 16), the second one was set at the beginning of the plateau phase (after 6 h, *n* = 16) and the third one at the point at which the first cells reached the maximum of the first derivative, after 2.5 h (wells A1–A8) and 3.5 h (Wells B1–B8), respectively (*n* = 16). After reaching the respective endpoints, the cells were washed twice with 400 µL DPBS solution (Merck, Darmstadt) and stained with hematoxylin and eosin. Pictures of the cell covering gold electrodes (*n* = 10 per well, 490,874 µm² surface each) of the ~22 h and 6 h dilution experiments were taken (Leica dmi8, Lasx software, Wetzlar). The cells were counted by selecting each picture section (Incscape 1.0) with a gold electrode of the same size, marking every cell covering the electrode. Any cells that only partially did so were counted if they were located in the upper and left section of the electrode. Finally, the size of the gold electrode was extrapolated for the whole well as homogenous cell distribution was expected. Furthermore, the counted cell number was compared to results of the ECIS R scripts and to the seeded cell number (*n* = 32).

### 2.4. IPEC-J2 Ethanol Treatment

Different behaviors in cell attachment, cell–cell interaction and cell proliferation were exerted by the addition of ethanol (EtOH), which is known to induce apoptosis and cell death in cultured cells above certain concentrations. After a five-hour attachment phase of the IPEC-J2 cells, the medium was removed and replaced by 400 µL of the treatment solution. For treatment solutions, 1% or 5% EtOH concentrations, respectively, were chosen depending on the existing literature [39,40,41,42]. The aim was to provoke (sub)lethal effects on the cells to uncover differences in growth behavior using the R scripts. The experiment was performed with four wells per run (quadruplets, two wells on each 8-well dish) and repeated five times in different weeks for 20 replicates per treatment in total.

### 2.5. ECIS Data Analysis with the Developed ECIS R Scripts

All the ECIS datasets were acquired with the ECIS software (Applied Biophysics, v1.2.186.0, US, Troy), exported as csv-documents (“selected wells/time”) and further saved in the .xslx format. All additional statistical and nonstatistical manipulations of the growth curves were performed with the R software (www.r-project.org, last accessed on 3 August 2021) and the following packages: readxl (.xlsx data import), segmented [33] (segmented regression), splines (base R) and minpack.lm [43] (nonlinear regression). All the R scripts and the “Examples” tutorial can be found in the author’s Github repository (https://github.com/anspiess/ECIS; last accessed on 3 August 2021) as well as in Supplemental Data 1 and offer the functionality described in Table 1.

### 2.6. Growth Curve Models, Area under the Curve (AUC) and Further Statistics

Three curve models were implemented as scripts for further calculations: an interpolating cubic smoothing spline model (spl; implemented in base R), a four-parameter logistic model [30,31,32] (log) and a segmented regression model [33] (seg). The logistic model, often used for ELISA [31], qPCR [30] and growth [32], is parametrized as *c* + (*d* − *c*)/(1 + exp(*b*(*x* − *e*)), where *c* indicates the lower asymptote, *d* the upper asymptote, *b* the slope and *e* the point of inflection. In principle, ECIS data should be normalized with normECIS prior to using this curve model. Segmented regression aims to estimate a new regression model with broken-line relationships defined by the slope parameters and the number of knots (breakpoints) where the linear relation changes. The number of knots of each segmented relationship is defined via the npsi argument. If npsi = 3, the growth curve should be divided into the baseline (one knot), growth (one to two knots) and plateau (one knot) regions, where each knot separates two adjacent linear segments.

The following values can then be derived from these curves using the parECIS function: *x* and *y* values of the first and second derivative maxima (spl, log), *x* value for 10% increase in impedance (spl, log), *x* values and slopes at 10%, 20% and 50% increase in impedance (seg), lower and upper asymptotes of given impedance (log), point of inflection and its slope (log) and baseline value/intercept and slope of all the segments with their nodal points (seg). A more detailed overview can be found in Supplemental Data 1, Chapter 14.

A further and frequently used method for curve comparison is the area under the curve (AUC). For instance, time-dependent blood plasma concentration of a drug is frequently described using the AUC for displaying an overview of the substance and its elimination over time [45,46,47,48,49]. In the ECIS case, the AUC is the integral of all impedance values *Z_i_* at each timepoint *t_i_*. It is feasible to use the AUC in situations where reduction in growth behavior over time is suspected but cannot be interpreted by specific timepoints like the maximum of the first derivative. The AUC can be evaluated with the intECIS function based on the sintegral function of the Bolstad2 package [44]. It takes a vector of *x* and a corresponding set of positive *y* = *f*(*x*) values and evaluates the AUC according to Simpson’s rule [50]:

$$\int_{a}^{b}f\left(x\right)dx≅h/3\left\{f\left(a\right)+f\left(b\right)+2\left[f\left(x_{2}\right)+f\left(x_{4}\right)+\ldots +f\left(x_{n-2}\right)\right]+4\left[f\left(x_{1}\right)+f\left(x_{3}\right)+\ldots +f\left(x_{n-1}\right)\right]\right\}$$

where the approximated numeric integral of any function *f*(*x*) in the range from *a* to *b* is given by the right-hand side formula for all *f*(*x*_1_) to *f*(*x*_n−1_) values.

## 3. Results

### 3.1. Curve Fitting and Further Statistics

Based on the growth model described above [13], the implemented three fitting models (spl, log, seg) were specifically designed to allow them to adapt to structural curve variations. Depending on the type of the curve, one or more models can be selected according to the chosen cell type, seeding concentration and treatments. All these experimental parameters influence the shape of the growth curve, resulting in a variety of curve trajectories that can differ with respect to baseline length, curve smoothness, slope and plateau length/linearity (Figure 1). It is therefore highly recommended to use the model that fits optimally the given curve type as determined by visual criteria or goodness-of-fit measures such as root-mean-square error (RMSE), residual variance (RV) or information criteria such as the Akaike information criterion (AIC). Whereas the spl model adapts particularly well to highly irregular curve situations, the log model can be taken if the curves exhibit an approximately sigmoidal structure. For the seg model, the best results are often achieved with 3–10 knots for ECIS growth curves with largely linear subregions, but this parameter can be fine-tuned.

**Figure 2:**
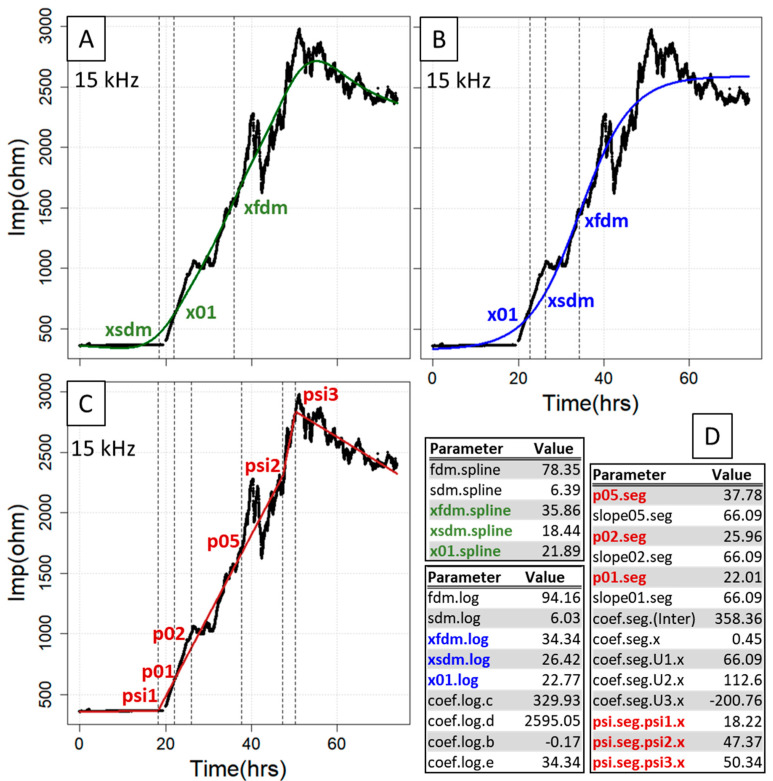
Essential curve features extracted from the fitting procedure for the smoothing cubic spline fit (**A**), logistic regression (**B**) and segmented regression (**C**). Shown are those parameters that pertain to abscissa values (time) at certain curve locations (FDM, SDM, 0.1/0.2/0.5× range, segmentation knots), where the complete fit parameter output for the three methods is depicted in (**D**). hrs = hours, Imp = impedance.

After the fitting procedure, all the *x* values generated with parECIS display the timepoint in hours, so they are particularly suitable if samples are to be taken at standardized times in subsequent experiments. Of special value among the curve points extractable from both the spl and log model are the first and the second derivative maxima as well as the interpolated *x* value at 10% increase of impedance (Figure 2A,B). The first derivative maximum indicates the point of largest impedance change on the gold electrode within the complete time interval. Its *x* value can be used when searching for the timepoint after which covering of the gold electrode becomes progressively slower or if it is assumed that there are differences in the speed at which the gold electrode is covered, in which case expedited coverage usually coincides with lower *x* values and higher slope. For the second derivative maximum constituting the point of inflection, it can be expected that all growth rates progressively decline from thereon. Moreover, the *x* value at 10% increase of impedance enables one to analyze possible changes at an early stage of the growth curve (transition of the baseline to the exponential phase). Finally, the log model extracts the averaged baseline (lower asymptote) and plateau (upper asymptote) impedance (parameters coef.log.c and coef.log.d), where it is important to note that the former can slip significantly below the actual baseline level if only defined by a few datapoints. In this case, it is possible to perform *a priori* normalization within (0, 1), which is automatically recognized by fitECIS. In contrast, the seg model can be used to not only obtain the interpolated *x* values for 10%, 20% and 50% increase of impedance, but also the knots (segment change points) from the fit, where the 10% and 20% values largely represent the growth behavior at the beginning or middle of the growth curve, and the 50% value a later change in growth behavior, for instance, at the onset/termination of a strong treatment (Figure 2C). If the number of knots is user-adjusted, finer division can be achieved, but care must be taken to not oversegment the curve. All the estimated parameters are provided for all three methods simultaneously as a sensible and compact table output (Figure 2D).

### 3.2. IPEC-J2 Dilution Experiments and Cell Counting on ECIS Dishes

**Figure 3:**
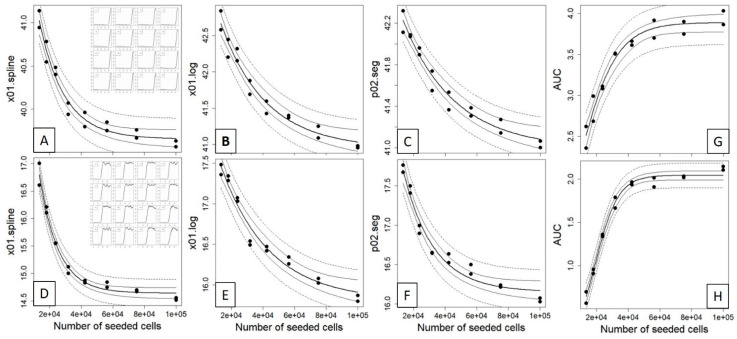
Nonlinear correlation between the seeded cell counts and the time estimated from the 0.1 quantile value of a spline (**A**,**D**), four-parameter logistic (**B**,**E**) and the 0.2 quantile value of segmented regression (**C**,**F**) of time vs. impedance (compare Figure 2 and Supplemental Data 2) for a 6 h (upper panel) and a plateau phase (lower panel) experiment, see insets. An exponential decay model *y* = (*y*_0_ − *y*_b_) × exp(−*kx*) + *y*_b_ was fitted to the data and displayed as fitted values (bold line), 95% confidence interval (thin line) and 95% prediction interval (dashed line). For both datasets, the area under the curve (AUC) was calculated for the complete time scale ((**G**); 0–46.9 h) and for a subset ((**H**); 0–20 h) and fitted with exponential growth model *y* = *y*_max_/(1 + exp(*a* + *bx*)). The corresponding fitted parameters and goodness-of-fit measures for both models are given in Table 2.

After having established the extractable curve features of the three different fitting methods, we investigated and evaluated whether different initially seeded cell counts would impact the time position of these curve features in a correlated manner (i.e., right shift with decreasing cell counts). To do so, we conducted a dilution experiment with seeded cell concentrations of 100,000, 75,000, 56,250, 42,188, 31,641, 23,731, 17,798 and 13,348 cells performed in duplicates over three endpoints (2.5 h, wells A1–A8; 3.5 h, wells B1–B8; 6 h, ~22 h, plateau; compare Supplemental Data 2, “Curve Data”). Here, we could clearly observe a correlation between the seeded cell count and the *x* value at defined impedance for cells that reached the plateau phase after 6 h (Figure 3A–C) and ~22 h (Figure 3D–F) at the estimated values for x01.spline, x01.log and p02.seg. However, for all of these three curve points and contrasting classical dilution series, the correlation between the seeded cell counts and the reference curve points was highly nonlinear and resembled an exponential decay model, which indeed fitted accurately the data with low root-mean-square error (RMSE) and residual variance (RV) (see Table 2). For these two timepoints, the AUC was inversely correlated to the reference curve points (Figure 3G,H) and followed an exponential growth model with decreasing AUC at increasing dilutions.

**Figure 4:**
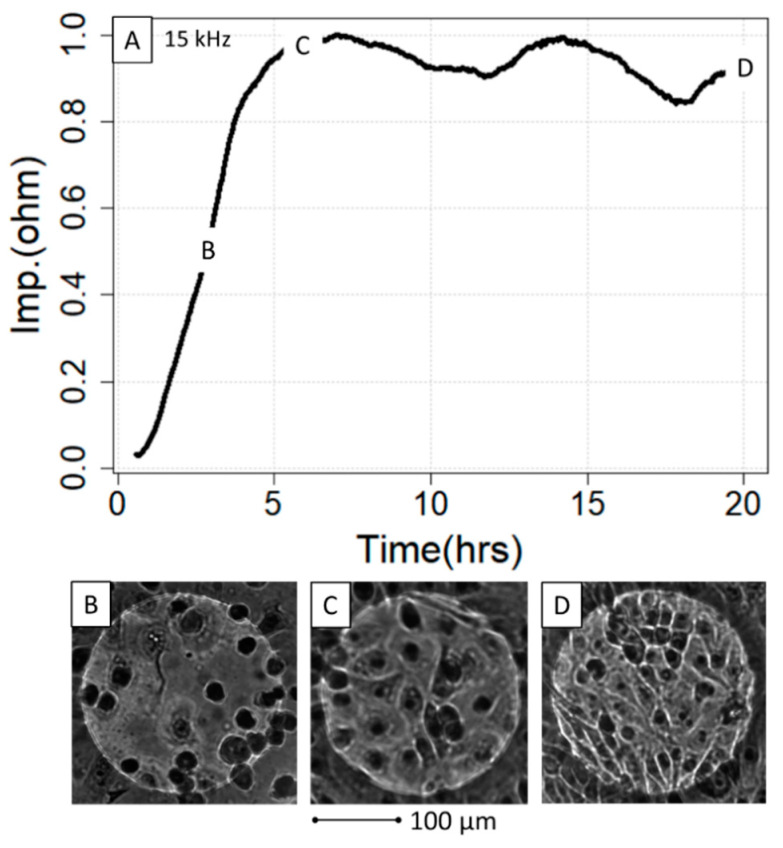
ECIS growth curve in relation to the IPEC-J2 density on the gold electrodes. The growth curve (**A**) was microscopically investigated at three different timepoints, 2.5 h (**B**), 6 h (**C**) and 22 h (**D**), with regard to cell density and interaction. Note that although (**C**,**D**) are in the plateau phase, the micrographs indicate significantly different cell density without impacting the impedance. hrs = hours, Imp = impedance.

Contrasting this, the results from the 2.5 h/3.5 h experiment did not provide sufficient curve points to be amenable to the analysis with the three fitting methods (Figure S1A). Here, we chose to exploit the maximum achieved impedance as the criterion, which, with respect to the seeded cell counts, could be modeled best with a quadratic model (Figure S1B) as based on Akaike weights [51] comparing linear, quadratic and logistic models (Figure S1C). For the comparison of seeded cells versus impedance, the ECIS growth curve of dish A was cut after 2.5 h so that a time span of a 2.5 h growth phase could be used for both dishes (wells A1–A8, wells B1–B8) as shown in Figure 4A. Finally, it is important to note that the maximum impedance could not be modeled well with the actual number of cells microscopically counted on the electrodes, i.e., at timepoints 2.5 h (dish A) and 3.5 h (dish B). When trying to investigate the association between electrode cell coverage and exerted impedance, it became apparent that the plateau phase value can be constant even at different densities of attached cells, reinforcing the observation that both are not connected in a linear fashion (Figure 4A–D; counted cells in Supplemental Data 2, “Cell Counts”).

### 3.3. Ethanol Treatment of IPEC-J2 Cells

**Figure 5:**
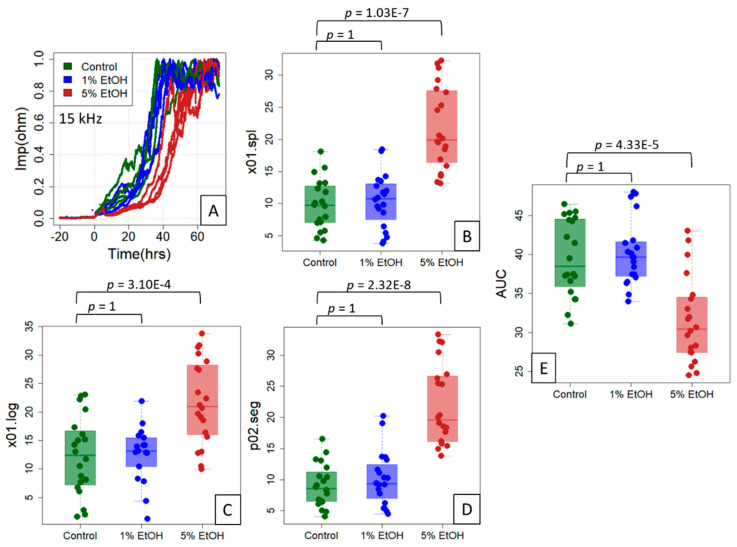
Reference points for the ethanol treatment experiment on IPEC-J2 cells. The cells were untreated (control) or treated with 1% or 5% ethanol (EtOH) (typical data shown in (**A**)) in an experimental setup of five independent experiments of four replicates each. Using the three location indices “Time at 10% spline” (x01.spline; (**B**)), “Time at 10% logistic fit” (x01.log; (**C**)) and “Time at 20% segmented regression” (p02.seg; (**D**)) on (0, 1)-normalized data (inset), a clear right shift of the impedance curve at 5% ethanol is evident. (**E**) The area under the curve (AUC) estimates of the complete time scale for all 20 replicates. A Welch (unequal variance) *t*-test, Bonferroni-corrected for multiple testing, was used to obtain *p*-values for the two group comparisons, control/1% EtOH and control/5% EtOH.

In the last step, we validated our models using ethanol-treated IPEC-J2 cells as it became apparent in the course of processing the corresponding ECIS data that a deviation in growth behavior was detectable, particularly at an early timepoint (Figure 5A). In all three models (spl, log, seg), the cells treated with 5% ethanol reached the x01.spline, x01.log and p02.seg points on the time axis significantly later when compared to the control cells (Figure 5B–D; *p* < 0.001). However, and as expected [39,42], no effect was evident at the treatment with 1% EtOH. Similar to the results described in Section 3.2, the AUC correlated inversely with all of the three curve reference points by being significantly smaller at 5% EtOH (Figure 5E; *p* < 0.0001).

## 4. Discussion

In this work, we developed and tested a set of R scripts that are useful for scientists working with ECIS growth curves, in which the data can be imported, processed, fitted to different curve models, plotted and quantified for later evaluation. These scripts facilitate the fitting of ECIS curves and subsequent reference points extraction for quantitation, which can be quite cumbersome when the researcher is not proficient in curve fitting.

We demonstrated in a real-life cell culture experiment on IPEC-J2 dilution and ethanol treatment data that defined curve reference points obtained from three implemented fitting methods correlate well and are nonlinear with the amount of initially seeded cells, but less so with the counted number actually residing on the electrode surface. These observations let us conclude that ECIS impedance for this frequency is primarily defined by epithelial closure (confluence) on the gold electrode and not the absolute cell number per se [24]. Hence, conclusions about cell numbers should therefore always be made by taking into account the cell line and the number of seeded cells; however, it is possible that cell lines with fewer changes in their individual morphology may show a stronger correlation between impedance and the counted cell number. Here, the shape of the cell, migration and similar parameters also influence the impedance level [27,37,38]. Furthermore, the frequency should always be taken into account when interpreting ECIS growth curve evaluations, where frequencies below 10,000 Hz are usually more suitable for characterizing cell–cell interactions and higher frequencies enable the actual coverage of the gold electrode to be monitored, which is likely the case here as the data were generated with the impedance of 15,000 Hz [38].

With the performed ethanol treatment experiment, we were able to show that differences in growth behavior between treated and non-treated groups can be resolved with our R scripts, and that based on this, practical timepoints for additional experiments can be chosen. Our established analysis pipeline can be used for diverse applications, for example, to quantitate the effect of any chemical, toxicological or pharmacological compound. Together with the appropriate measurement frequency, it offers the possibility to conduct large-scale quantification of cell junction and/or adherence-disrupting agonists [52].

For further experiments with other cell lines and treatments than the ones conducted in this work, it has to be ensured that the best-performing fitting method and appropriate curve feature is selected to optimally model cell-specific differences in growth behavior. Here, it must be noted that in the context of an ECIS experiment, a change in impedance is a multifactorial mixture of cellular and physical effects as the behavior of a cell population in toto is considered. For instance, there appear to be several explanations for the level of the plateau phase, where the passage number of cells can come into role when displaying time against resistance, possibly from a limitation in “barrier-forming capabilities” [38]. We found a difference in the impedance value at the plateau phase level at different cell seeding densities and, although considerately subtler, in EtOH-treated and non-treated cells. We assumed that for our ECIS data, the maximum impedance level must be present at complete coverage of the gold electrode; however, as we were unable to find a correlation between the counted cell numbers and the impedance values in the plateau phase, we conclude that the level of plateau phase impedance is not dependent on the number of cells present at 100% confluence. While this parameter should not affect the statistics for, e.g., *x* values of the maximum of the first derivative, we chose to use normalized data (normECIS) for calculations of the AUC to ensure comparability between different final impedances values. Importantly, when presenting ECIS impedance data as the AUC, it must be decided up to which timepoint it is calculated; for instance, it should be decided to select the time at which the last growth curve reaches the plateau phase or where each individual curve reaches its individual entry into the plateau phase. In addition, calculation of the AUC up to a further and previously determined timepoint can also be considered when a change in growth behavior is expected only until the maximum of the first derivative. However, in this case, caution is advised if IC_50_ is to be determined on the basis of the AUC [53].

## 5. Conclusions

With our newly developed toolset of R scripts, we can improve the evaluation and quantitation of growth curves generated with ECIS hardware and make them fully comparable between different experiments. Various applications are conceivable in the context of cell research, especially with regard to the investigation of cell growth behavior, cell proliferation, cell differentiation and determination of sampling or treatment times. Although the evaluation of ECIS growth curves alone by means of our supplied scripts is possible, further conclusions can be drawn for other impedance measurement methods in experimental questions relating to cell growth.

In addition, it can be assumed that the selected evaluation methods can also be transferred to the evaluation of other types of growth curves (for example, the growth of bacterial or algae populations) as long as the evaluated populations show similar growth behavior to the cell one and some key points are similar (for example, a similar number of measurement points and similar growth curve progression). Further research and adaption of the R package is needed to pursue this point.

## Figures and Tables

**Table 1:** Analysis functions implemented as R scripts for the manipulation and quantitation of ECIS curves.

Function Name	Brief Description
getECIS	Import of raw ECIS .xlsx files into the R data frame
plotECIS	Plotting of ECIS datasets in a variety of ways
cutECIS	Cutting of time ranges from ECIS datasets
delECIS	Deletion of specific wells from ECIS datasets
selECIS	Selection of ECIS wells to form a new dataset
addECIS	Combination of two or more different ECIS datasets
baseECIS	Subtraction of the baseline value of each specific well
normECIS	Normalization of ECIS datasets to (0, 1)
intECIS	Numerical integration of the area under the curve [44]
fitECIS	Calculation of different curve models and features from ECIS datasets as described in Section 2.6
parECIS	Getting all the parameters acquired by fitECIS
extECIS	Removal and extension of the leading region of ECIS data
anoECIS	Identification and deletion of outliers of an ECIS dataset
setECIS	Setting of a start point of an experiment to zero

**Table 2:** Parameters and goodness-of-fit measures derived from fitting the exponential decay model (spl, log, seg) and the growth model (AUC) to the data in Figure 3.

	Cut, 6 h				Cut, Plateau			
	Spl	Log	Seg	AUC	Spl	Log	Seg	AUC
***Y*_0_**	42.86	43.71	42.87		21.26	18.38	19.28	
***Y*_b_**	39.65	40.93	40.98		14.64	15.8	16.15	
***k***	0.000063	0.000035	0.000031		0.000083	0.000033	0.000052	
***Y*_max_**				3.89				2.04
***a***				0.471				2.56
***b***				0.00008				0.00014
**RMSE**	0.089	0.11	0.075	0.1	0.096	0.097	0.099	0.056
**RV**	0.0097	0.015	0.0068	0.013	0.011	0.012	0.012	0.0037
***R*^2^**	0.967	0.964	0.966	0.96	0.984	0.969	0.967	0.99
**AIC**	−24.11	−17.26	−29.71	−20.07	−21.5	−21.33	−20.74	−39.18

## Data Availability

Supporting data can be can be found in the author’s Github repository (https://github.com/anspiess/ECIS; last accessed on 3 August 2021).

## Supplementary Materials

The following are available online at https://www.mdpi.com/article/10.3390/s21165286/s1, Figure S1. Use of the maximum impedance max(Imp) as a criterion when a complete curve trajectory is not available. In this 2.5 h scenario, the maximum impedance of the baselined data at 15 kHz (A) was regressed against the number of seeded cells (B). AIC values and the derived Akaike weights indicate that a quadratic model outperforms a linear and logistic model (C). Supplemental Data 1. Overview of the 15 ECIS-related functions that are implemented as R scripts downloadable at https://github.com/anspiess/ECIS (accessed on 3 August 2021). For each function, an overview of its theoretical background, functionality and use of function arguments is provided together with graphical examples. Supplemental Data 2. Raw curve data (“Curve Data”) and cell counts (“Cell Counts”) used for the analysis and creation of Figure 2, Figure 3, Figure 4 and Figure 5 in this work.
