# Supplementary material for: Modelling and Differential Quantification of Electric Cell-Substrate Impedance Sensing Growth Curves
Source: Sensors (Basel). 2021 Aug 5;21(16):5286. doi: 10.3390/s21165286 (PMC8401457; doi:10.3390/s21165286)
Supplement: Supplementary file 1 [file sensors-21-05286-s001.zip › Supplemental Data 1.pdf]

<u>Supplemental Data 1: ECIS Analysis Functions</u>

<u>Content</u>

1. Packages (library)
2. Uploading of ECIS datasets (`getECIS`)
3. Plotting ECIS curves (`plotECIS`)
4. Baseline correction (`baseECIS`)
5. Cutting ECIS curves (`cutECIS`)
6. Deleting ECIS wells (`delECIS`)
7. Selecting ECIS wells (`selECIS`)
8. Adding ECIS datasets (`addECIS`)
9. Extending ECIS curves (`extECIS`)
10. Outlier correction (`anoECIS`)
11. Set zero point (`setECIS`)
12. Normalize (`normECIS`)
13. Numerical Integration by Simpson's Rule (`intECIS`)
14. Fit functions (`fitECIS`)
15. Output (`parECIS`)

1. Required Packages (library)

library(minpack.lm)
library(segmented)
library(splines)
library(readxl)

2. Uploading of ECIS datasets (`getECIS`)

getECIS <-function (file, freq = NULL)

Preparation: Choose desired time and modifications in the ECIS software (Figure S1) -> download dataset from ECIS-software: file -> export data ->selected wells/time -> save as CSV.file -> save CSV.file as XLSX.file

If multiple frequencies are available: either use [*optional]* function `freq = …` or selectable frequencies will be displayed during running the `getECIS` function → „Available Frequencies [Hz]: […] Select one, please:" → type choosen frequency as number (e.g. 16000)

In the uploaded ECIS dataset, resistance and capacitance (if available) will be deleted.

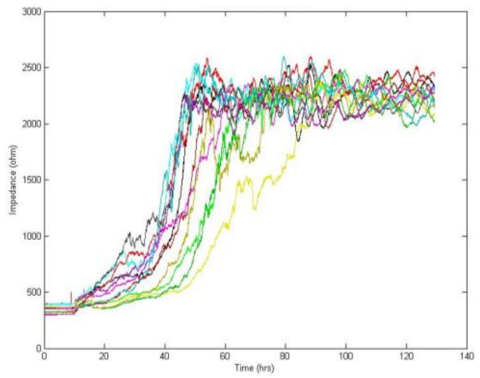

Figure S1: ECIS curve, ECIS-software output

- **file** → **"**filename**.xlsx**" or "filename**.csv"**
- [optional] **freq** → frequency e.g. 16000

3. Plotting ECIS curves (`plotECIS`)

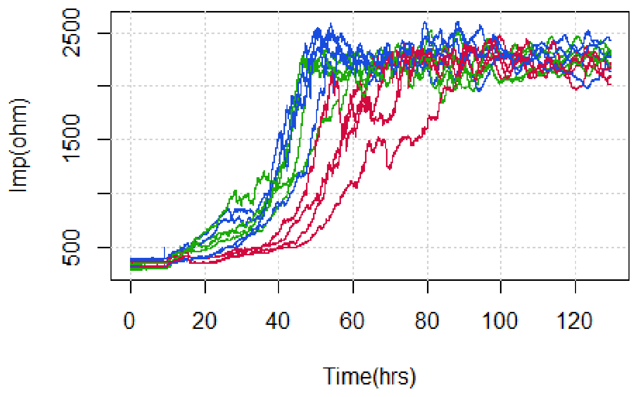

Figure S2: 1003-A2- plotECIS function – fit = “none“, type = “single”

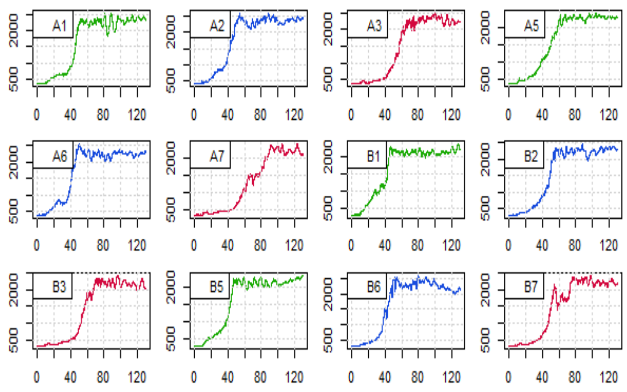

Figure S3: plotECIS function, type = “matrix”

plotECIS <- function (ecis, which = NULL, style = c (“matrix”, “single”), fit = c (“none”, “spl”, “log”, “seg”), col = NULL, legend = TRUE, cex.legend = 1, par = NULL, …)

Plotting ECIS datasets (either as single plot => Figure S2 or multiple plots => Figure S3)

- **ecis** → name of ECIS dataset (imported with `getECIS`)
- [optional] **which = NULL** → if online parts of one dataset should be plotted, related to wells (e.g. which = c(1,2,3)
- [optional] **style** = **NULL** (= “matrix”) or either of the two:
  - ➔ “matrix” = multiple plot
  - ➔ “single” = all curves in one plot
- [optional] **fit = NULL** → = “none”, or any of the following **fit** =
  - ➔ “none”*:* ECIS impedance curve
  - ➔ “spl”: smoothing spline model
  - ➔ “log”: 4-parameter logistic model
  - ➔ “seg”: segmented regression spline model
- [optional] **col = NULL** → color of plots (e.g. col = c(“skyblue”, ”darkred”))
- [optional] **legend = TRUE** → show a legend with well names, or alternatively a character vector of length(ecis) with legend names to be shown
- [optional] **cex.legend = 1** → legend font size
- [optional] **par = NULL** → a list of parameters for plot setup, as in par(…)
- [optional] **…** other parameters to be passed to `plot.default`

4. Baseline correction (`baseECIS`)

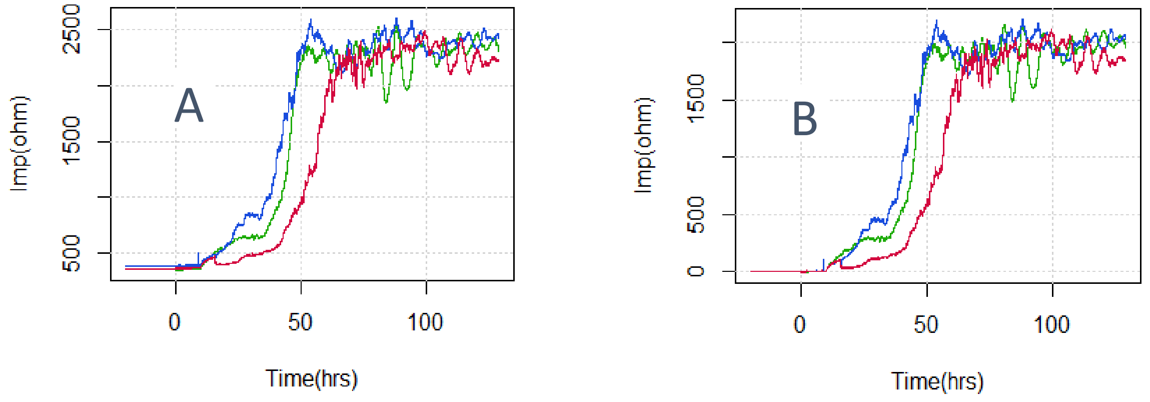

Figure S4: A) Raw data with a baseline of ~400, B) and a baseline of 0 after baseline correction

baseECIS <- function(ecis, type = c("mean", "median"), window = 2)

Each ECIS well will have some kind of individual background noise that should stay stable over time (follow ECIS instructions: only stable, if e.g. cysteine treatment was done or ECIS plates were incubated with culture medium before) => Figure S4A. To remove this individual baseline for each well, run ECIS with culture medium but without cells, before seeding cells. This baseline should stay stable and can be subtracted for each individual well with the `baseECIS` function => Figure S4B.

- **ecis** → name of ECIS dataset (imported with `getECIS`)
- [optional] **type =** → choose one:
  - → "Mean"
  - → "Median"
- [optional] **window = 2** → time defined as "baseline", e.g., the time ECIS was running with culture medium but without cells (in hours; e.g. window =2 → 2 hours)
- use before `extECIS` or `setECIS`

5. Cutting ECIS curves (cutECIS)

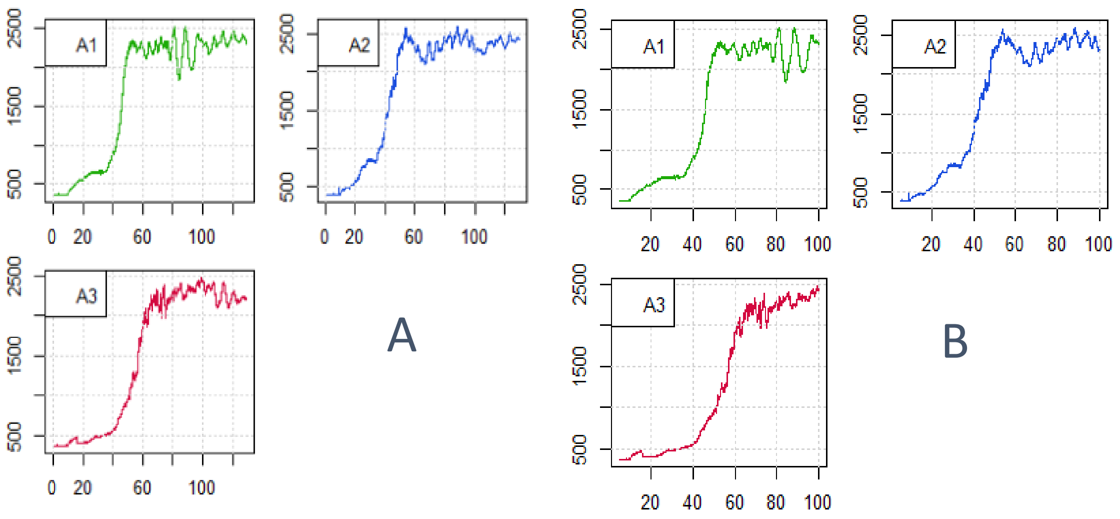

Figure S5: A) Without cut, B) and with cut at 5h and 100h

cutECIS <- function (ecis, min = NULL, max = NULL)

If only part of data is needed: cut at desired time point(s).

- **ecis** → name of ECIS dataset (imported with `getECIS`)
- [optional] **min = NULL** → time in h (e.g. `min = 5.3`); data before this time (x value, in hours) will be cut off (CAVE: is NOT replacing new starting time with starting time = 0, see `setECIS`)
- [optional] **max = NULL** → time in h (e.g. `min = 5.3`) // data after this time (x value, in hours) will be cut off

6. Deleting ECIS wells (delECIS)

delECIS <- function(ecis, which)

Remove one or more wells of an ECIS dataset that are not desired.

- **ecis** → name of ECIS dataset (imported with `getECIS`)
- **which** → refers to well order (e.g. which = c(2, 3))

7. Selecting ECIS wells (selECIS)

selCIS <- function(ecis, which)

Select and extract one or more wells of an ECIS dataset. This will give a new ECIS dataset.

- **ecis** → name of ECIS dataset (imported with `getECIS`)
- **which** → refers to well order (e.g. which = c(2, 3))

8. Adding ECIS datasets (addECIS)

addECIS <- function (…)

Combine two or more ECIS datasets
Time in hours (x value length) can differ afterwards!

- **…** → **(**name of ECIS-dataset1**,** name of ECIS-dataset2, …**)** → will concatenate all datasets

9. Extending ECIS curves (extECIS)

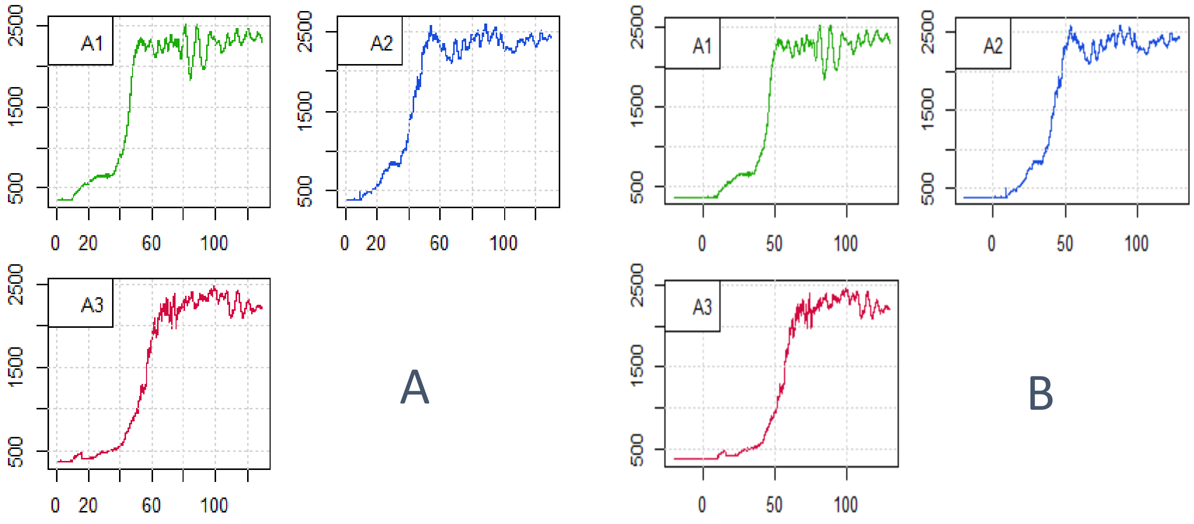

Figure S6: A) Without extension, B) and with 20 hours extension at curve beginning

extECIS <- function(ecis, extend = NULL, n = 10)

Extends ECIS curves in either direction, i.e. additional time points/impedance values can be added to the beginning (longer baseline) => Figure S6B or end (longer plateau phase) of the curve, based on the median of a defined range of cycles.

- **ecis** → name of ECIS dataset (imported with `getECIS`)
- **extend = NULL** → choose until which x value (time in hours) the curve should be extended in either direction. For instance, if the curve starts at 0 and the baseline should be extended to -10, use `extend = -10`. If the plateau ends at 120, set `extend = 150` to extend the data range to 150
- [optional] **n = 10** → choose how many time points (single points, not hours!) should be used at the beginning or end to calculate the median value for extension

10. Outlier (anomaly) correction (anoECIS)

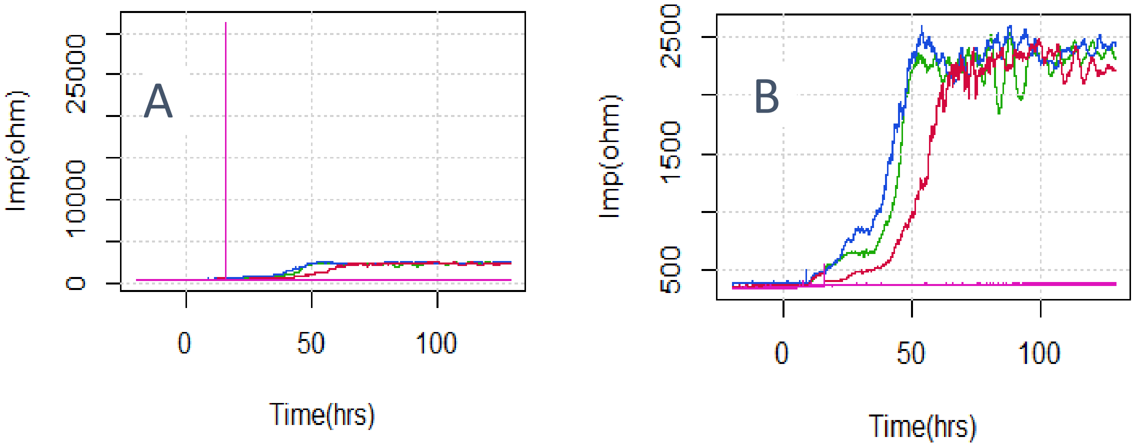

Figure S7: A) Technical outlier in control group with pure medium (pink), B) and after outlier correction

anoECIS <- function(ecis, spar = 1, fac = 10)

Outliers can occur in ECIS curves in several ways (e.g. technical errors or a forgotten break before removing the dish) => Figure S7A. If the reason for these outliers is known and the growth behavior was not influenced by the error, these outliers can be clearly identified by the function and replaced => Figure S7B. To do so, a smoothing spline curve, residues and interquartile ranges (IQR) are calculated. The result is then multiplied with a factor `fac` (y(spline) + `fac` * IQR). Values above or below will be defined as outliers and replaced by y(spline).

- **ecis** → name of ECIS dataset (imported with `getECIS`)
- **spar = 1** → smoothing parameter, typically (but not necessarily) in [0,1], should be optimized empirically
- **fac =10** → factor, multiplied with IQR, also to be optimized

11. Set zero point (setECIS)

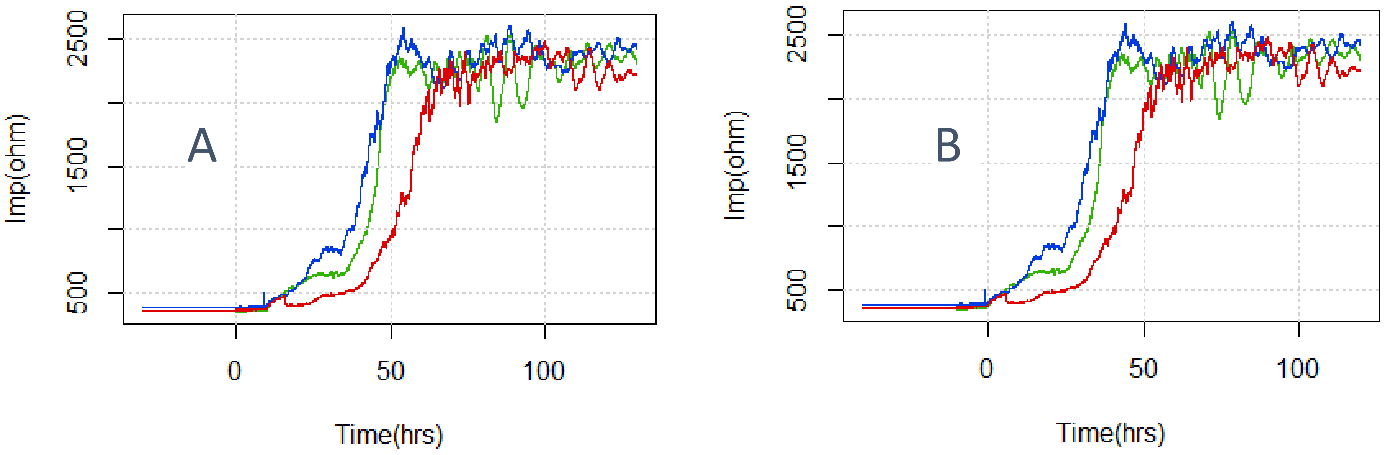

Figure S8: A) Zero point at 0h and B) set zero point to 10h

setECIS <- function(ecis, time = NULL, trim = FALSE)

Only ECIS data without negative x values (time) can be imported by the `getECIS` function. This function will set one defined x value (time in hours) to zero, for example at certain time points like seeding time of the cells or at the time point of treatment. This procedure might also be necessary after using extECIS.

- **ecis** → name of ECIS dataset (imported with `getECIS`)
- **time = NULL** → time in hours that should be set to zero (e.g. `time = 0.5`)
- [optional] **trim = FALSE** → if `TRUE`, x values (time in hours) before `time` will be cut off

12. Normalize (normECIS)

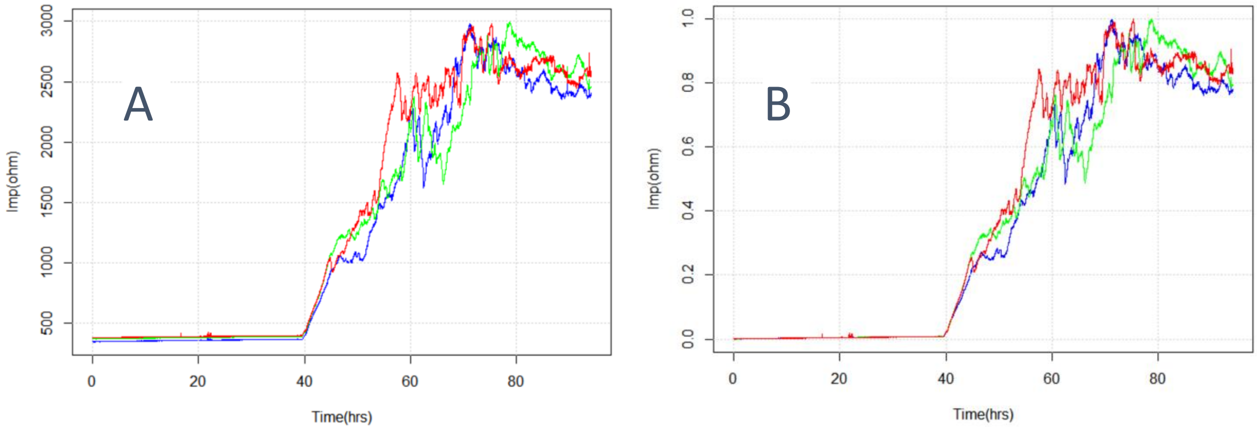

Figure S9: A) Raw data and B) normalized to [0, 1]

normECIS <- function(ecis)

Often, analyses work better if the ECISdata => Figure S9A is normalized to [0, 1] => Figure S9B prior to any downstream methods such as fitting (smoothing spline, segmented regression, log).

- **ecis →** name of ECIS dataset (imported with `getECIS`)

13. Numerical Integration by Simpson's Rule (intECIS)

intECIS <- function(ecis, min = NULL, max = NULL)

Based on the `sintegral` function of the Bolstad2 package. Takes a vector of *x* values and a corresponding set of positive f(*x*) = *y* values and evaluates the area under the curve according to Simpson's Rule[1]:

$$\int_a^b f(x)dx \cong$$

$$\cong \frac{h}{3}\{f(a) + f(b) + 2[f(x_2) + f(x_4) + \cdots + f(x_{n-2})] +$$

$$+4[f(x_1) + f(x_3) + \cdots + f(x_{n-1})]\},$$

For setups that deliver very heterogeneous curves, it might be feasible to use `baseECIS` or `normECIS` before using `intECIS`, in order to restrain the derived integral to a common *y*-scale.

- **ecis →** name of ECIS dataset (imported with `getECIS`)
- **min = NULL →** lower integral boundary *a*, if not defined, min(*x*)
- **max = NULL →** upper integral boundary *b*, if not defined, max(*x*)

[1]Numerical Integration. Gordon K. Smyth, Encyclopedia of Biostatistics (2005), John Wiley & Sons, Hoboken, NJ, USA.

14. Fit functions (fitECIS)

fitECIS <- function(ecis, which = NULL, nknots = 200, npsi = 3)

fitECIS will fit three different models to an ECIS growth curve: a smoothing spline model (”spl”, *R* base), a four-parameter logistic model (“log”, library *minpack.lm*) and a segmented regression model (“seg”, library *segmented*).

- **ecis** → name of ECIS dataset (imported with `getECIS`)
- [optional] **which = NULL** → refers to well order (e.g. which = c(1, 2))
- [optional] **nknots = 200** → refers to the number of knots in the smoothing spline model
- [optional] **npsi = 3** → refers to the number of knots in the segmented regression model

14.1 Smoothing spline model

This is the interpolating cubic smoothing spline model from *R* base. We have found 200 knots (nknots = 200) to give a good approximation to ECIS curves, without losing too much curve structure but not overfitting. However, this parameter can be tweaked.

14.2 Four-parametric logistic model

This is classical four-parameter logistic model that is used frequently to model growth[2], ELISA[3] and qPCR[4] curves:

$$\mathrm{y} = \mathrm{c} + \frac{d - c}{1 + e^{(b(x-e))}}$$

where *c* = lower asymptote, *d* = upper asymptote, *b* = slope and *e* = point of inflection. If the data has been normalized by `normECIS` prior to fitting, this is recognized and the more simpler [0, 1]-bounded model

$$\mathrm{y} = \frac{1}{1 + e^{(b(x-e))}}$$

is employed.

[2]Seber GAF, Wild CJ. Nonlinear Regression (2003), John Wiley & Sons, Hoboken, NJ, USA.
[3]Ratkowsky DA, Reedy TJ. Choosing near-linear parameters in the four-parameter logistic model for radioligand and related assays. Biometrics. 1986 Sep;42(3):575-82.
[4]Liu W, Saint DA. Validation of a quantitative method for real time PCR kinetics. Biochem Biophys Res Commun. 2002 Jun 7;294(2):347-53.

14.3 Segmented regression model

Given a linear regression model, segmented regression aims to estimate a new regression model with broken-line relationships[5]. A segmented relationship is defined by the slope parameters and the break-points where the linear relation changes. The number of breakpoints of each segmented relationship is fixed via the `npsi` argument, where a defined number of knots must be provided. We have set `npsi = 3` as this models the baseline region (one knot), the growth region (one to two knots) and the plateau region (one knot) fairly well.

[5]Muggeo, V.M.R. (2008) Segmented: an R package to fit regression models with broken-line relationships. R News 8/1, 20–25.

15. Output (parECIS)

parECIS <- function(fit)

- **fit →** an object obtained from `fitECIS`

Output: a list with the results of the three fitting methods

Mathematical model	Derived parameter	Output value
smoothing spline model<br>4-par logistic model	first derivated maximum (*y* value)	fdm.spline<br>fdm.log
smoothing spline model<br>4-par logistic model	second derivated maximum (*y* value)	sdm.spline<br>sdm.log
smoothing spline model<br>4-par logistic model	first derivated maximum (*x* value)	xfdm.spline<br>xfdm.log
smoothing spline model<br>4-par logistic model	second derivated maximum (*x* value)	xsdm.spline<br>xsdm.log
smoothing spline model<br>4-par logistic model	*x* value at 10% increase in impedance	x01.spline<br>x01.log
4-par logistic model	Lower asymptote of given impedance (*y* value)	coef.log.c
4-par logistic model	Upper asymptote of given impedance (*y* value)	coef.log.d
4-par logistic model	Slope of curve at point of inflection	coef.log.b
4-par logistic model	Point of inflection (*x* value)	coef.log.e
segmented regression	*x* value at 10%, 20% and 50% increase in impedance	P01.seg, p02.seg, p05.seg
segmented regression	slope at 10%, 20% and 50% increase in impedance	slope01.seg, slope02.seg, slope05.seg
segmented regression	baseline value / intercept (*y* value)	coef.seg.(Intercept)
segmented regression	slope of segment 1	coef.seg.x
segmented regression	slope of segments 2-4	coef.seg.U1-U3.x
segmented regression	*x* at nodal points (knots) 1 to 3	psi.seg.psi1-3.x
