# Supplementary figures and images for: Modelling and Differential Quantification of Electric Cell-Substrate Impedance Sensing Growth Curves
Source: Sensors (Basel). 2021 Aug 5;21(16):5286. doi: 10.3390/s21165286 (PMC8401457; doi:10.3390/s21165286)

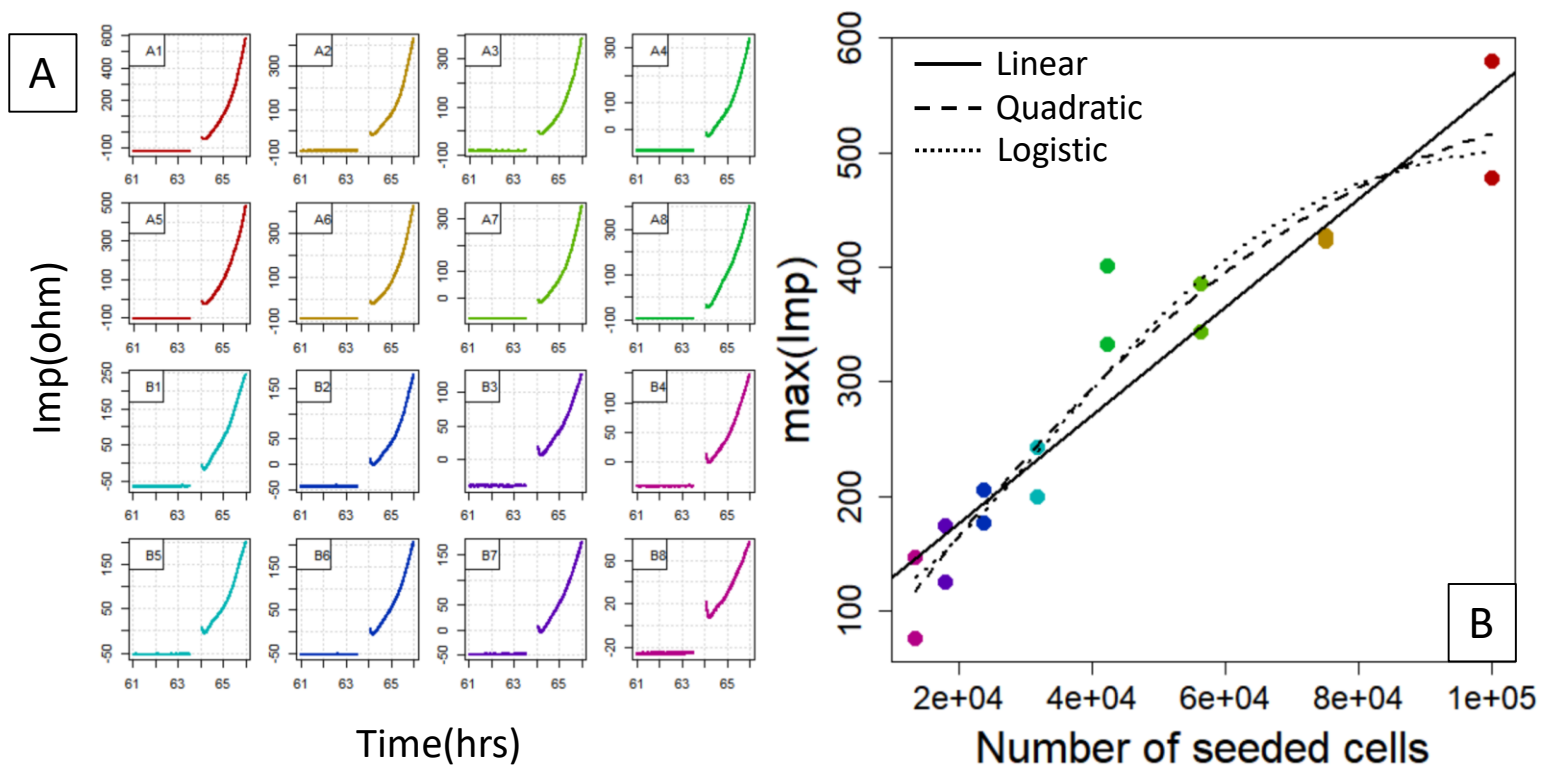

Figure S1

**C**

|                  | AIC   | Weights |
|------------------|-------|---------|
| <b>Linear</b>    | 173.6 | 0.13    |
| <b>Quadratic</b> | 170.5 | 0.61    |
| <b>Logistic</b>  | 172.3 | 0.26    |

Supplement: Supplementary file 1 [file sensors-21-05286-s001.zip › Figure_S1.pdf]
